# Development of an online pan-European Integrated Pest Management Resource Toolbox

**DOI:** 10.12688/openreseurope.14679.1

**Published:** 2022-06-06

**Authors:** Mark Ramsden, Margherita Furiosi, Paolo Debenedettis, Isidora Stojacic, Marta Mendes, Nicolas Munier-Jolain, Tito Caffi

**Affiliations:** 1 RSK ADAS Ltd., ADAS Boxworth, Cambridgeshire, UK; 2 DIPROVES, Catholic University of the Sacred Heart, Via E. Parmense 84, Piacenza, 29122, Italy; 3 BioSense Institute, University of Novi Sad, Dr Zorana Djindjica 1, Novi Sad, 21000, Serbia; 4 Consulai, Consultoria Agroindustrial Lda, Rua da Junqueira, 61 G, Lisboa, 1300-307, Portugal; 5 INRAE, UMR University/INRA/ENVN, Dijon, France

**Keywords:** Integrated Pest Management, survey data, resource toolbox, multi-actor

## Abstract

The IPM ***WORKS*** IPM Resource Toolbox (Toolbox) has been developed as an interactive, online repository of integrated pest management (IPM) resources. Populated with high priority resources for farmers and their advisors during the project, its structure enables additional resources added over time. The repository is a public interactive website, available to anyone looking to access, understand, and implement IPM. Built on an open-source content management system, the toolbox is designed to require minimal post-production site maintenance and support, while being easily expanded to integrate resources from future initiatives.

To ensure an efficient but comprehensive website design, population, maintenance, a survey of target user needs was conducted. Internal and external IPM stakeholders indicated the relative importance of key requirements such as practical information about diseases and pests’ management and economic thresholds. The resources were explained in different languages, with images, divided by topics, with the possibility to find additional details and accessible by smartphone. Feedbacks and answers from the survey, carried out across Europe in multiple languages, by different stakeholders provided the key elements and foundation for the IPM Resource Toolbox website development and specification.

## Plain language summary

How can integrated pest management (IPM) strategies be accessed by users and applied on farm? IPM ***WORKS*** IPM Resource Toolbox is an online repository of IPM resources. A survey about stakeholders’ expectations and use of IPM toolbox was carried out with expected users (grouped in different categories as Farmers, Agronomists, Researchers, Developers, Policy makers/advisors, Retail organizations and others) to understand their requirements, and to collect their opinions about toolbox utility and how it can be developed and managed.

## Introduction

Intensive use of pesticides has led to many problems such as resistance to agro-chemicals, reduction of natural enemies’ and non-target organisms’ population, and rise of chemical residues in final products ( Ali *et al.,* 2021), all contributing to a general increase of production costs. Modern agriculture strives to achieve more sustainable farming systems, with less reliance on pesticides. The challenge of reducing inputs applied in fields and reducing the negative effects on the environment, while retaining a high quality and quantity of the final yield, preserving, or increasing farmers’ incomes is complicated but achievable ( Barzman *et al.,* 2015; Rezaei *et al.,* 2019; Rossi *et al.,* 2012). To face these issues integrated production was developed, aiming to reach a high-quality production by giving the priority to ecological methods. In this context integrated pest management (IPM) aims to achieve a sustainable crop protection against harmful organisms, keeping the use of chemical pesticides at levels that are economically and environmentally acceptable. In Europe IPM became mandatory in 2014 ( Directive 2009/128/EC) and the European Union is progressively banning or limiting the most dangerous pesticides for human health and environment ( Dara, 2019; Hillocks, 2012; Lamichhane *et al.,* 2016a; Lefebvre *et al.,* 2015; Rossi *et al.,* 2012; Way & van Emden, 2000). IPM is based on eight principles and considers all processes and components characterizing the agro-system, to reach a long terms sustainable crop production and protection ( Barzman *et al.*, 2015; Lamichhane, 2017; Rossi *et al.,* 2019).

Agricultural production inevitably becomes more complicated over time, as new innovations in plant breeding, machinery, application technology, management systems, require more time and knowledge to identify suitable approaches and integrate them into existing approaches. Increasing the system complexity therefore increases the benefits of methods, tools, and thresholds to decide whether, when, where and what kind of intervention should be carried out, based on updated and detailed information about crops and diseases in a specific context ( Rossi *et al.,* 2012). Different tools are already available to help farmers in managing pests, such as warning service, on site device and decision support systems (DSS). Even if these tools provide updated information about weather conditions and forecast, crop growth and risks of key pests/disease at regional or farm level ( Gent *et al.,* 2011; Lamichhane *et al.,* 2016a; Rossi *et al.,* 2012), DSSs and other resources still have a limited uptake among farmers due to a range of actual and perceived barriers, such as farmers not being aware of what resources are available, or resources (such as pest forecast systems) focusing on individual pests while farmers are facing multiple infestations across the farm. IPM resources should satisfy users’ need in the real field context and demonstrate the economic advantage of their adoption.

To fully implement IPM principles a holistic approach is needed, involving the application of different strategies to optimize pests’ control, considering economic and environmental thresholds ( Barzman *et al.*, 2015; Hillocks, 2012; Stenberg, 2017). However, in many cases the results of IPM research and innovation projects are difficult to be applied by farmers. They perceive IPM as time consuming, and the application of multiple control strategies against different pests is hard in the field context, where different crops require different interventions ( Ehler, 2006; Hillocks & Cooper, 2012; Stenberg, 2017). Farmers see IPM as a labyrinth of tools and the potential economic advantages are rarely clear ( Rossi *et al.,* 2019; Way & van Emden, 2000). A lack of knowledge and understanding of IPM principles can lead to increased confidence in pesticide efficacy, thus farmers’ education to IPM principles is crucial to make them able to evaluate pesticides, their effects, and make appropriate decisions ( Gent *et al.,* 2011; Hashemi & Damalas, 2011; Van den Berg & Jiggins, 2007). Without support, IPM has a limited success ( Ehler, 2006; Lefebvre *et al.,* 2015; Parsa *et al.,* 2014). Many European initiatives, such as IPMWorks (H2020, No. 101000339), IPMDecisions (H2020 No. 817617), and SmartProtect (H2020 No. 862563) are promoting the adoption of IPM strategies and available tools across Europe to achieve more sustainable farming and plant protection. These fixed term projects develop resources to support advance implementation of IPM towards ‘Holistic IPM’. Providing access to resource, training, technical support, and demonstrations of IPM in action and the consequence of their management on the environment improves uptake and implementation ( Cameron, 2010; Parsa *et al.,* 2014; Rezaei *et al.,* 2019). At present, IPM resources are spread across multiple projects and associated websites, and this is creating a barrier to access for farmers looking to improve IPM implementation across Europe. 

One objective of the IPMWORKS project is to develop a Resource Toolbox to provide easy access to IPM resources to different stakeholders, making IPM Resources widely accessible in a consistent format across Europe, promoting improved uptake of effective IPM tactics and strategies. This Toolbox is an interactive, online repository of IPM resources, populated with high priority resources for farmers and their advisors during the project, with further resources added over time. The repository is an interactive website with a dedicated search engine, accessed by both consortium members and the public. The website is built on an open-source content management system, requiring minimal post-production site maintenance and support to ensure longevity. This toolbox enables users to easily search, share, and discuss IPM resources. Resources will include training modules, results of demonstration trials, leaflets describing cost-effective IPM strategies, videos of farmers’ testimonies and demonstration events, training materials, DSS (from IPMDecisions), cultural control descriptions, and resource evaluation material. Resources collated in other EC funded projects can be readily integrated, such as for IPM resources within the ENDURE Information Centre and SmartProtect IPM Thematic Network platform.

To ensure an efficient but comprehensive website design a survey of target user needs was conducted. Internal and external IPM stakeholders indicated the relative importance of key requirements. Feedback from these surveys, carried out across Europe, provided the foundation for the website specification.

## Methods

**Figure 1.**
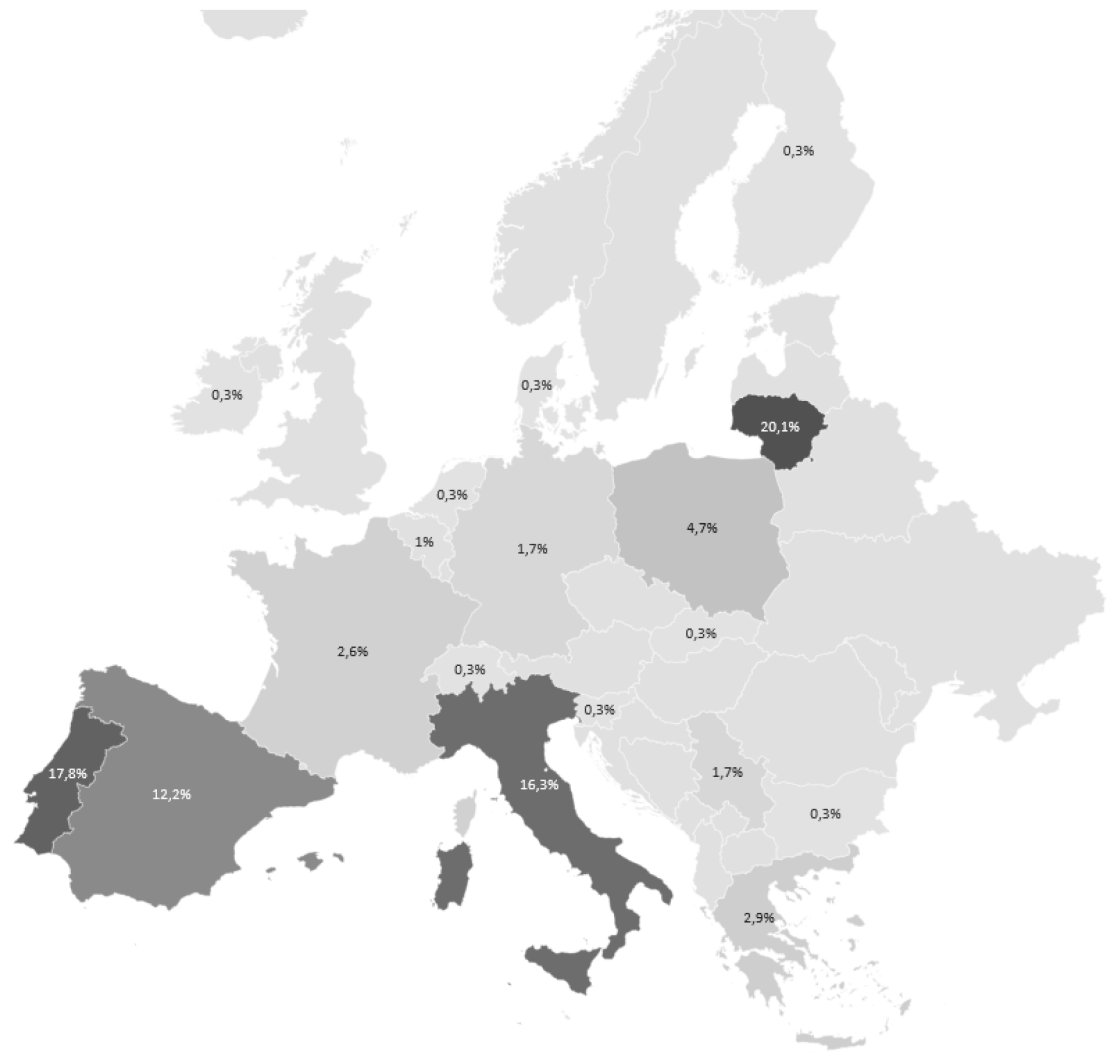
Response share to the anonymous survey across Europe (n= 343: different shades on the map show different share of respondents.

The anonymous survey was carried out between November 2020 and January 2021 with potential end users. The survey was open access, available in multiple languages and promoted by project partners in both IPM ***WORKS*** and IPM *Decisions* ( Figure 1) through social media channels. The survey was addressed to farmers and advisors, but anyone could answer questions. Potential IPM Resource Toolbox users were categorized into one of the following groups: Farmers, Agronomists, Researchers, Developers, Policy makers/advisors, Retail organizations and others ( Table 1). Respondents didn’t receive any reward for participation.

**Table 1.** Description of target groups for the IPM Resource Toolbox.

Target Group	Description	Total number of answers
**Farmers**	Individuals working on farms, responsible for enacting and/or making agricultural decisions.	119
**Agronomists**	Individuals working with/for farmers in an advisory role, providing agricultural support, advice and/or making decisions on behalf of farmers. Individuals may be acting independently, or as part of an industry organization.	110
**Researchers**	Individuals undertaking agricultural research, including academic and/or applied research, as well as innovation and development activities. Researchers/ higher education students/ research and higher education institutions	79
**Software developers**	Individuals creating or expanding software services within the agricultural sector.	7
**Policy advisors/** **makers**	Individuals responsible for supporting, advising and/or making policy decisions.	9
**Retail organisations**	Individuals working within food supply chains, including (for example) logistics, processing and retail.	8
**Other**	Individuals not directly involved in food production, retail, or policy development, e.g. Consumers, general public etc.	11

The study didn’t expect a specific sample dimension, and Microsoft Office Forms was used to create and share the survey. The survey wasn’t validated before use; collected data were validated, excluding no meaning data/answers. Firstly, the survey asks general information about users, such as the country where they live, which crop they are interested in, and to which group they belong. Almost 90% of respondents were farmers, agronomists and researchers, minimizing the risk of bias ( Table 1). Then, the survey asks about the actual approach they use for crop management, what they expect from IPM toolbox and what could encourage their use of toolbox. Finally, the survey asks what can be improved on the website and if they know some European projects. The survey collected mainly quantitative data, where users could select the answer among multiple choices or using a Likert scale. No personal data was collected in the survey, all responses were anonymous, and questions could be left blank, considering missing data as indifference or lack of opinion by respondents. The full questionnaire is available in the *Extended data* ( Ramsden, 2022b).

The data were managed to visualize responses from different stakeholder groups, expressing different opinions and expectations. Microsoft Excel was used to manage data and to create graphs and tables. Users were categorized into groups, knowing the number of respondents in each group and the total number of respondents, we calculate the percentage of participants belonging to each group.

## Results and discussion

**Figure 2.**
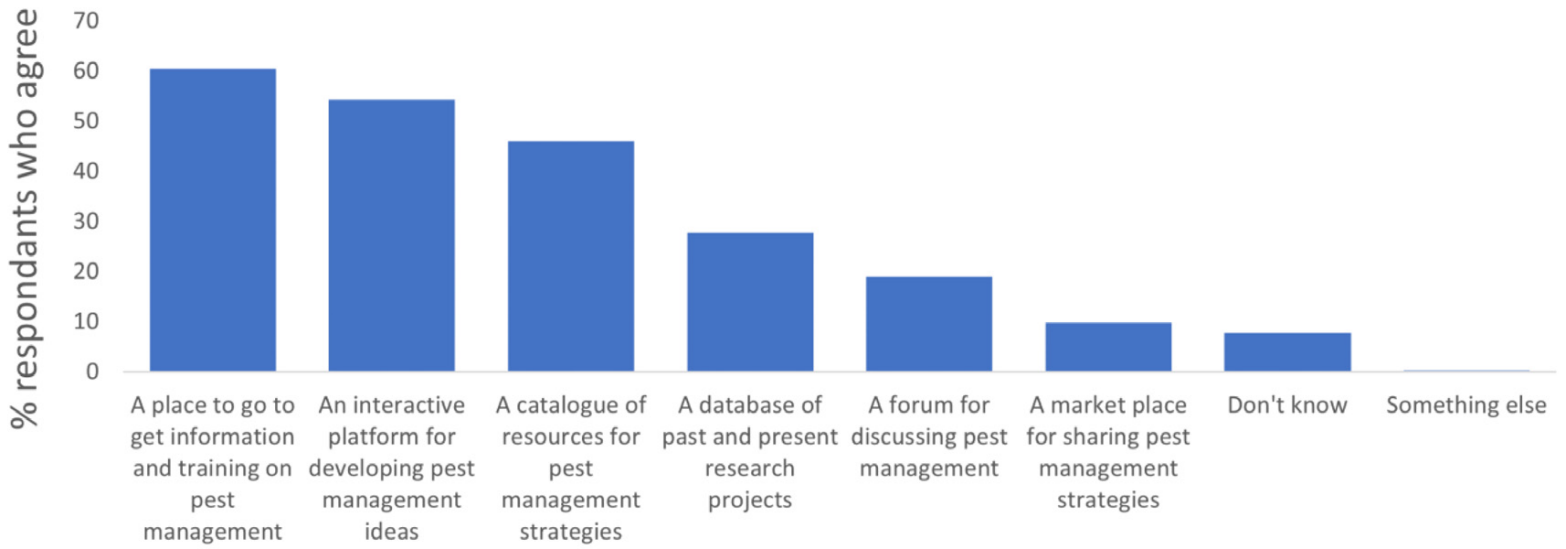
Share of end users who agreed with one (or more) technical characteristics that the ongoing IPM Resource Toolbox should fulfil.

Most users (60%) expected to be able to access information and training on pest management (60% of farmers and agronomists) ( Ramsden, 2022a). Most of them expected it to be an interactive platform (40% of farmers and agronomists and 70% of researchers), with a catalogue of resources for pest management strategies. There was less expectation across users that the platform would provide a database of projects, a forum for discussion, or a market for sharing strategies. Only few respondents weren’t sure what the Toolbox might offer or felt that it would offer something other than the proposed ideas ( Figure 2).

### Resources that would encourage the use of IPM Resource Toolbox

**Figure 3.**
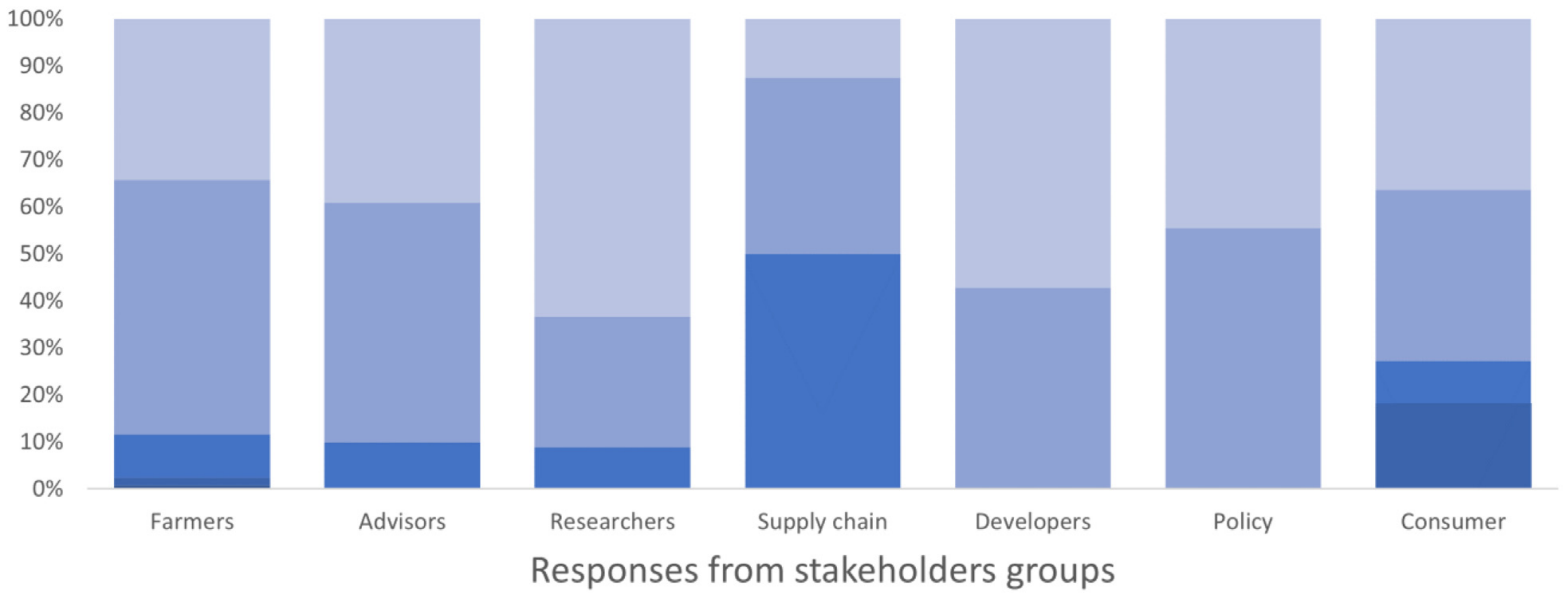
Degree of importance given to the description of IPM techniques and evaluation of their economic and environmental impacts by different stakeholders’ group, expressed as a percentage of the number of respondents in each group. The degree of importance ranged between 1 and 5 in the survey, where 1 corresponded to “not important” and 5 “very important”. The degree is represented by a blue colour scale, from darker blue for value 1 to lighter blue for value 5.

All users identified economic thresholds as a valuable tool for inclusion in the toolkit. Monitoring tool descriptions, economic and environmental details, pest forecasts, crop planning tools, and technical IPM guides were identified as especially important tools for inclusion. The description of IPM techniques and the evaluation of their economic and environmental impact were considered important especially by researchers, developers, policy makers, farmers, and advisors ( Figure 3). Training resources were also seen as important within the Toolbox, but more so by researchers than practitioners (farmers and agronomists). Testimonials and case studies weren’t seen as being important as tools that specifically detailed the technical elements of implementation and impacts.

### What users value when accessing online resources

**Figure 4.**
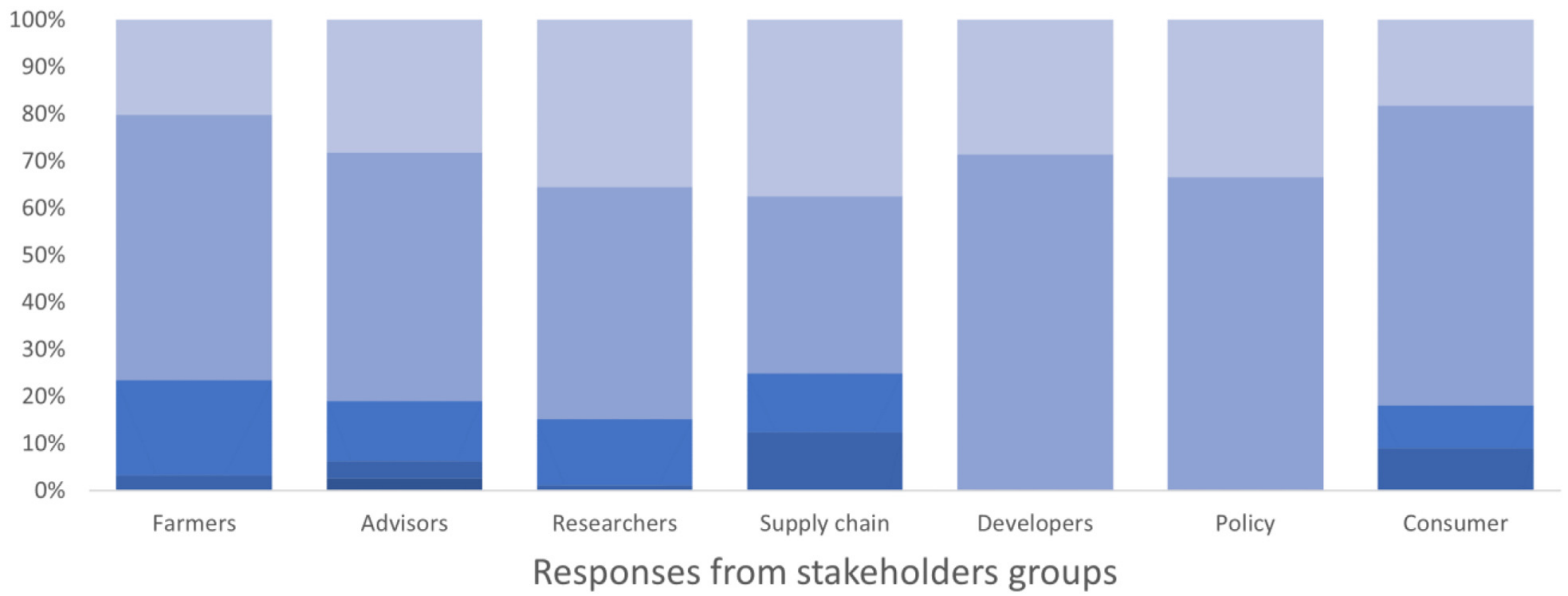
Stakeholders groups’ trust in content when they know who produced/shared it, expressed as a percentage of the number of respondents in each group. The degree of importance ranged between 1 and 5 in the survey, where 1 corresponded to “not important” and 5 “very important”. The degree is represented by a blue colour scale, from darker blue for value 1 to lighter blue for value 5.

**Figure 5.**
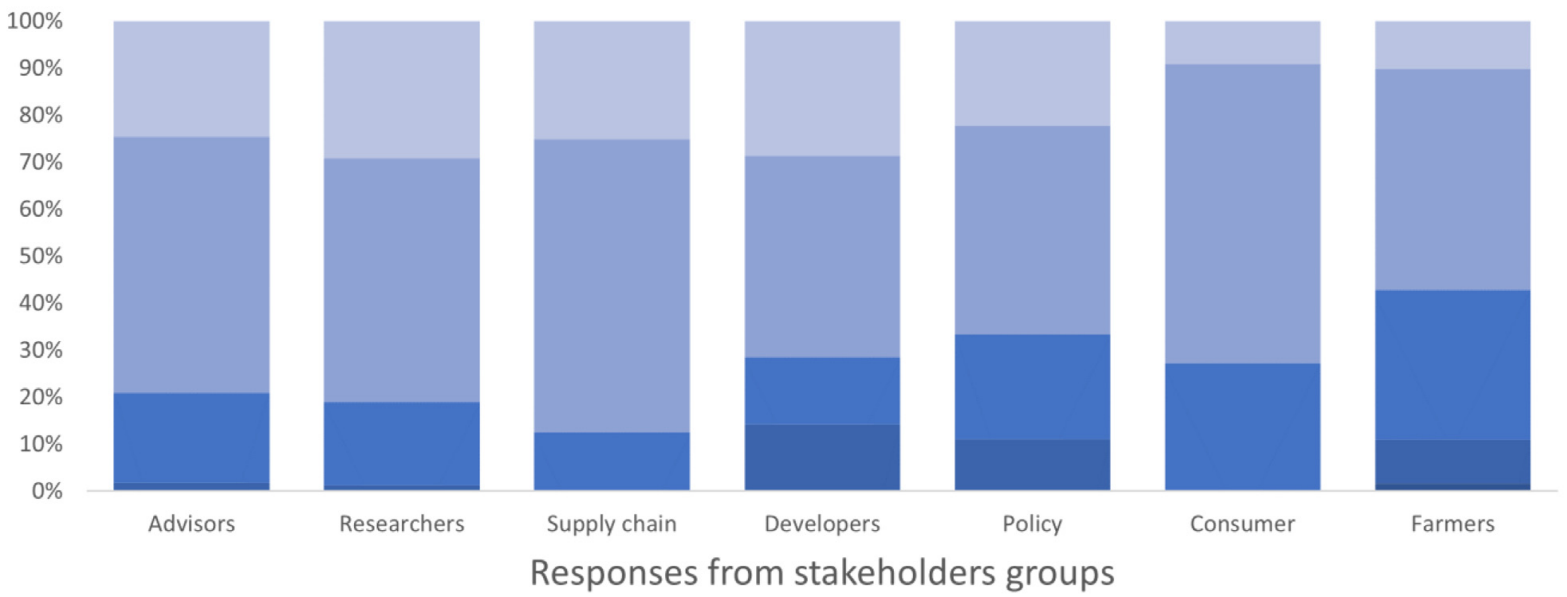
Preference to image-based content by different stakeholder groups, expressed as a percentage of the number of respondents in each group. The degree of preference ranged between 1 and 5 in the survey, where 1 corresponded to "I don't prefer image-based contents" and 5 corresponded to "I prefer/want image-based contents". The degree is represented by a blue colour scale, from darker blue for value 1 to lighter blue for value 5.

All users identified being able to see the names of people who shared content as increasing their trust in the material ( Figure 4). Most users would like to access a lot of detail on a topic; advisors particularly valued access to details. All users preferred image-based content, especially researchers, developers, advisors, policy makers, and supply chain workers, compared to video, text or audio contents ( Figure 5). Some user types (supply-chain workers/other) preferred video content. No strong preference was found for text or audio-based contents.

### Stakeholder awareness of past/current related projects

**Figure 6.**
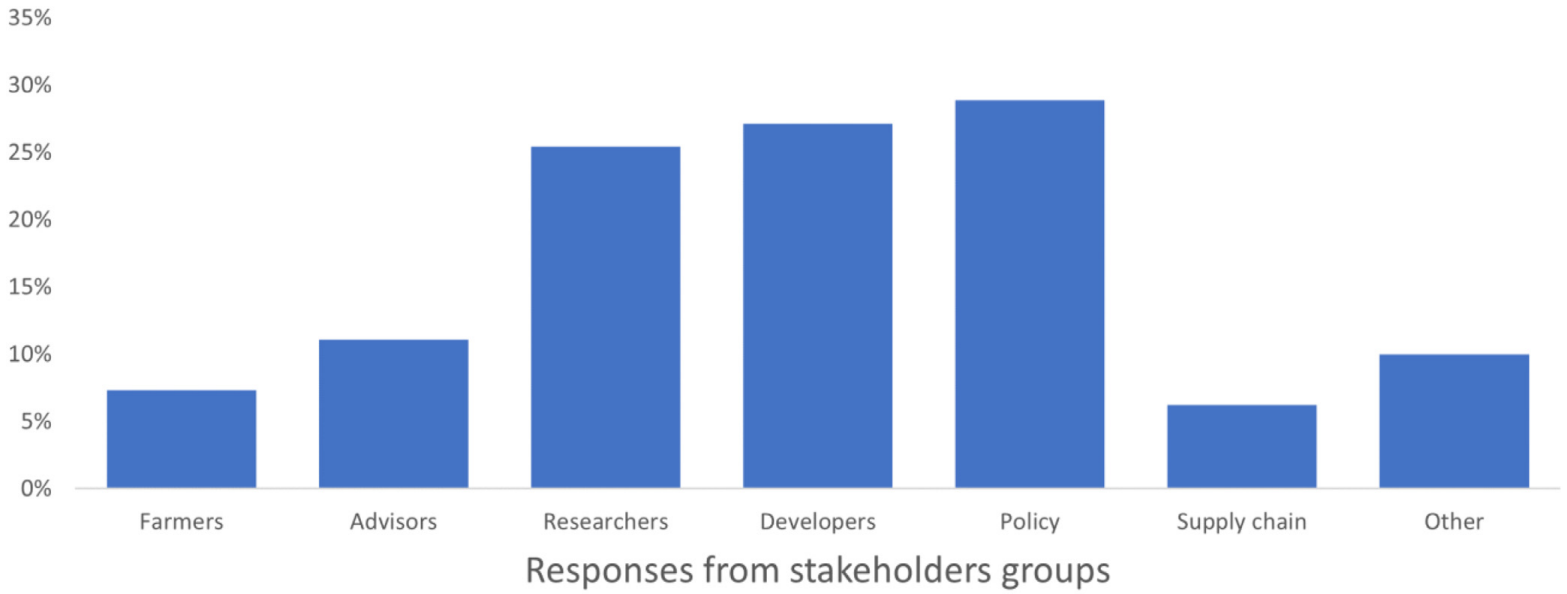
Average stakeholders’ awareness of past and current projects, expressed as a percentage on the number of respondents in each group.

Most respondents weren’t aware of the suggested projects. Researchers together with developers and policy makers were most aware of past and current projects, but among these groups only 25–30% of them know some projects. On the other hand, among farmers, advisors, supply chain workers, only few know of past and present projects (6–11%) ( Figure 6). This means that a deep revision in how projects’ topics, goals and results are communicated to a broad audience is needed. As suggested by Burbi & Rose (2016) and Lamichhane *et al.* (2018), internet and social media channels represent an opportunity to connect farmers and other stakeholders, with the possibility to create online communities to share information, experiences, innovations, and to attend congress or events promoting knowledge exchange.

### Additional comments

Stakeholders indicated some improvements to make the IPM toolbox more usable. Considering the accessibility, being a platform available across Europe, contents should be expressed in users’ native languages ( Barzman *et al.,* 2014; Grasswitz, 2019; Lamichhane *et al.,* 2016b), to be more understandable by farmers, because not all of them speak English. In fact, users asked to minimize text contents that require translation. Farmers have little time and they see IPM as time consuming ( Ehler, 2006; Lefebvre *et al.,* 2015; Rossi *et al.,* 2019), so they suggested the possibility to access the Toolbox directly from their smartphones to save time and to consult resources also when they are in fields. Contents should be clear, easy to find and tailored to each farm context, describing only the essential and providing recommendations. Information and resources should require minimal clicks to be found in the site, with contents divided into topics and expressed in the simplest way possible, especially if they are about technical aspects. Moreover, stakeholders would like to find easily links to other existing platforms and to have the possibility to read scientific articles about the main topics. Users that have access to scientifical papers, researchers’ results, or participate to events, are more likely to adopt IPM strategies ( McNamara *et al.,* 1991), but most of time scientifical results, articles or congress are not open to farmers, advisors etc. ( Hoffmann *et al.,* 2007). Considering the technical contents, final users suggested that they should cover a wider range of pests, diseases and beneficial organisms and they should follow the ongoing of the season, because farmers normally face much more issues compared to what is present in the platforms. Farmers need practical information, in fact toolbox should express technical content in a simple way, targeted to implementation, and minimizing theorical contents. If resources are context specific, as related to a region, a variety, or a soil type, it should be clarified.

Most users were interested in the current and future European projects, and they would like to receive news about these. Particularly, H2020 DiverIMPACTS project is developing a Toolbox to find the most relevant resources for users, according to their need, and to encourage the diversification in the agricultural sector for both technical aspects and sector development. Finally, about 96% of respondents were interested in finding out more about IPMworks online IPM Resources Toolbox, confirming the great attention that agricultural stakeholders deserve to research and innovation, especially in the last years ( Hoffmann *et al.,* 2007).

**Figure 7.**
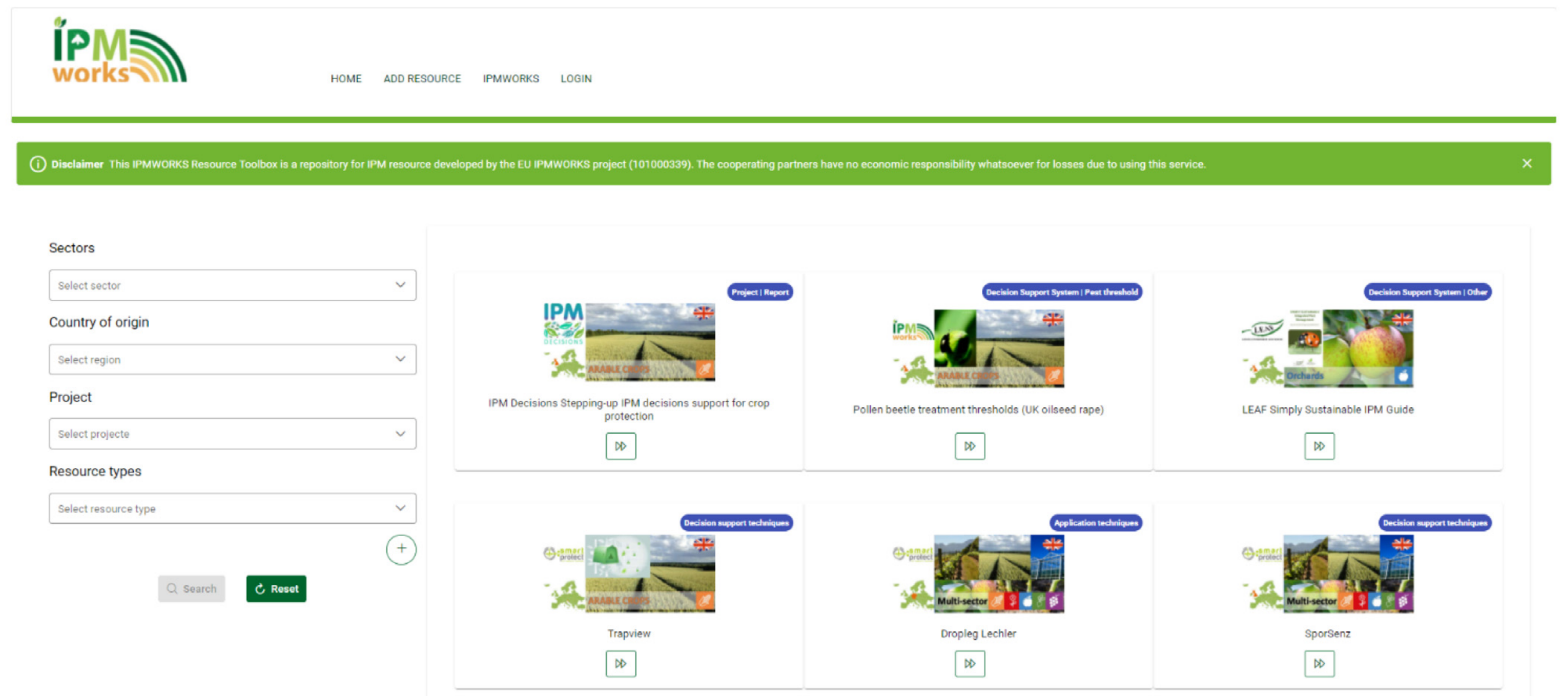
IPM resource Toolbox homepage.

The Toolbox was developed following the information collected during the abovementioned survey, following users’ suggestions and feedbacks. The IPM Resource Toolbox homepage ( Figure 7) was developed in an easy and clear way to allow search of resources, selecting one or more attributes from the following list: sector of interest, country of origin, project, resource type, specific crop or pest, and language. No login requirement was planned: on the contrary, the available resources are highlighted, and users can search them freely.

New resources can be added to the toolbox for free by the “add resource” tab ( Figure 7), filling in a short form with the required information, such as a description of the resource characteristics, the sector which the resource is referred to, the country of origin, relevant pests and crops, the resource type, the project title and, if it’s an European project, the grant number, and the language. The toolbox also gives the possibility to upload images and other resources, and to add further information as a link to the original website, DOI, institutional contacts, email, etc. All the uploaded information must be approved by a team member before being available for public.

**Figure 8.**
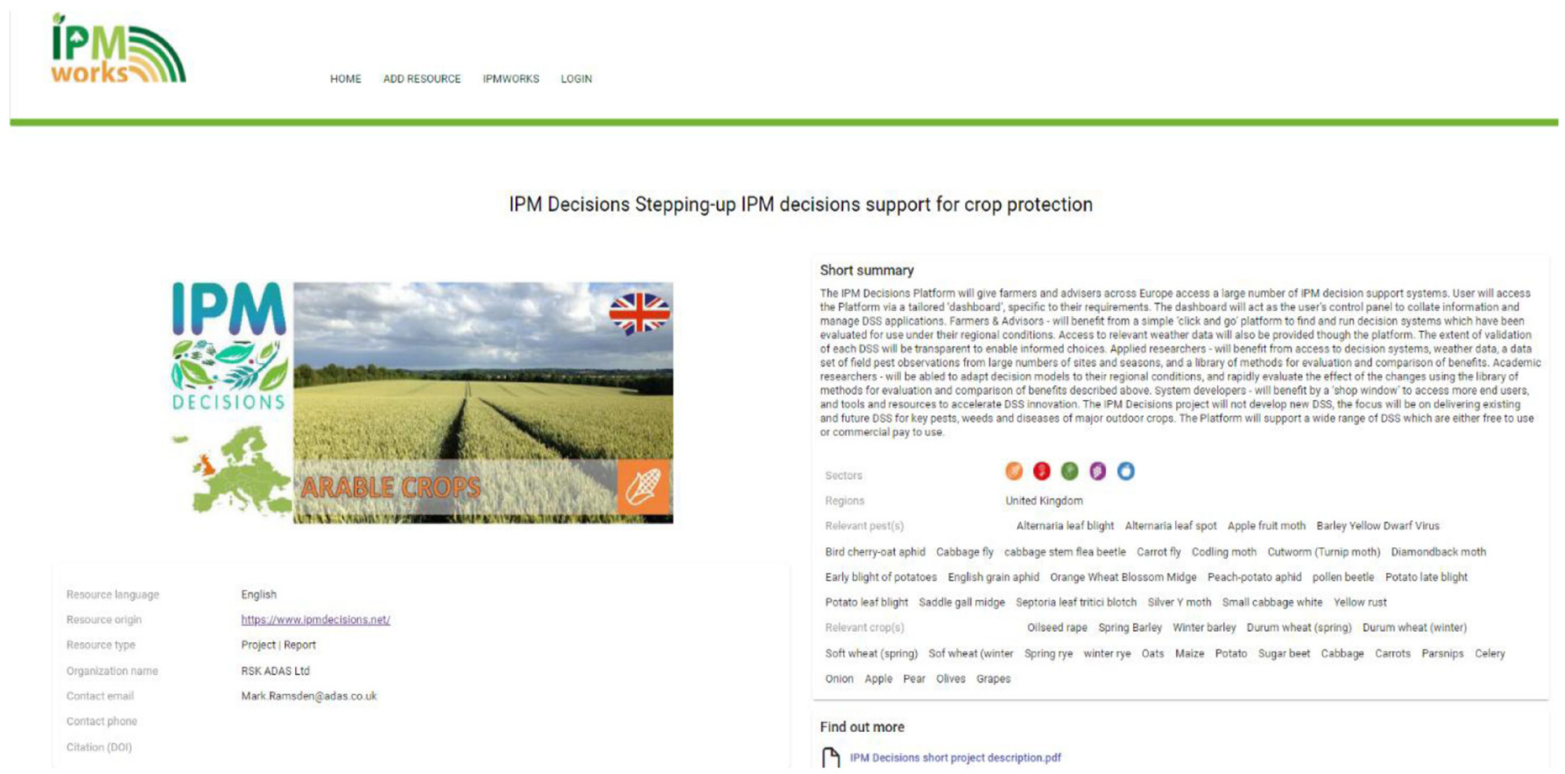
An example of available resource on the IPM resource Toolbox.

Once the resource of interest is found, users can click and see more details about the topic. An example is reported in Figure 8, where user can see the title of the topic, the resource origin, the producer with a contact to eventually ask for more information or clarification, a short summary of the resource, also indicating the sector to which is referred and a link to additional materials to deepen users understanding about the topic.

## Conclusions

Target users expect the Toolbox to provide quick access to descriptions of IPM resources, and associated training on how to implement those resources. They want the information to be easily accessible, but also be able to see further details wherever available. Users indicated a preference for image-based content, but other formats were all acceptable. Survey was the starting point to understand better what stakeholders need, to create a useful Toolbox, designed for users. The toolbox will be an important tool for spread the adoption of IPM principles across Europe.

The toolbox enables stakeholders to both access existing resources, and share their own outputs, with peers from across Europe.

## Data availability

### Underlying data

INRAE: IPMWorks H2020 No. 101000339 – IPM Resource Toolbox Survey raw data.

https://doi.org/10.15454/MGHARY ( Ramsden, 2022a).

This project contains the following underlying data:

- IPMWorks H2020 No.101000339- IPM Resource Toolbox Survey raw data.tab (Note: this brief report is based exclusively on answers related to the survey (however, the questionnaire contains several questions whose answers were not included in this study)

### Extended data

INRAE: IPMWorks H2020 No. 101000339 – IPM Resource Toolbox Survey Questionnaire. https://doi.org/10.15454/7YD5VL ( Ramsden, 2022b).

This project contains the following extended data:

- IPM Resource Toolbox Survey.pdf

Data are available under the terms of the Creative Commons Zero "No rights reserved" data waiver (CC0 1.0 Public domain dedication).

## Ethics and consent

The research was conducted according to requirements of ethics and integrity in Article 14 of the Model Grant Agreement of Horizon Europe. The research activity was complied with the ethics provisions set out in the Grant Agreement, particularly: highest ethical standards and applicable European law.

No personal data were collected, all data collected were anonymous. Ethics approval and consent were not required for this study.
