# Supplementary material for: Development of an online pan-European Integrated Pest Management Resource Toolbox
Source: Open Res Eur. 2022 Sep 22;2:72. Originally published 2022 Jun 6. [Version 2] doi: 10.12688/openreseurope.14679.2 (PMC10445940; doi:10.12688/openreseurope.14679.2)
Supplement: Peer review history

# Peer review: Reviewer response for version 2

**DOI:** 10.21956/openreseurope.16328.r30130

**Published:** 2022-10-06

**Authors:** Emilio Gil, Ramon Salcedo

**Affiliations:** 1 Unit of Agricultural Machinery Research, Department of Agri Food Engineering and Biotechnology, Universitat Politècnica de Catalunya, Barcelona, Spain

I made a review on the new version of the manuscript. From my side everything is OK and ready to be indexed.

Is the study design appropriate and does the work have academic merit?

Yes

Is the work clearly and accurately presented and does it cite the current literature?

Yes

If applicable, is the statistical analysis and its interpretation appropriate?

Not applicable

Are all the source data underlying the results available to ensure full reproducibility?

Partly

Are the conclusions drawn adequately supported by the results?

Yes

Are sufficient details of methods and analysis provided to allow replication by others?

Yes

Reviewer Expertise:

Full professor in Ag Engineering, focused in spray application technology and aspects related with environmental contamination

We confirm that we have read this submission and believe that we have an appropriate level of expertise to confirm that it is of an acceptable scientific standard.

# Peer review: Reviewer response for version 2

**DOI:** 10.21956/openreseurope.16328.r30131

**Published:** 2022-09-23

**Authors:** Ilias Travlos

**Affiliations:** 1 Laboratory of Agronomy, Department of Crop Science, Agricultural University of Athens, Athens, Greece

Authors have addressed the vast majority if the raised issues and consequently their paper can be accepted for indexing.

Is the study design appropriate and does the work have academic merit?

Yes

Is the work clearly and accurately presented and does it cite the current literature?

Yes

If applicable, is the statistical analysis and its interpretation appropriate?

Not applicable

Are all the source data underlying the results available to ensure full reproducibility?

Yes

Are the conclusions drawn adequately supported by the results?

Yes

Are sufficient details of methods and analysis provided to allow replication by others?

Yes

Reviewer Expertise:

Associate Professor of Agronomy & Weed Science

I confirm that I have read this submission and believe that I have an appropriate level of expertise to confirm that it is of an acceptable scientific standard.

# Peer review: Reviewer response for version 1

**DOI:** 10.21956/openreseurope.15856.r29681

**Published:** 2022-08-19

**Authors:** Ilias Travlos

**Affiliations:** 1 Laboratory of Agronomy, Department of Crop Science, Agricultural University of Athens, Athens, Greece

In general, the study is a comprehensive overview of the outcomes and activities within the IPMWorks H2020 project. The overall approach and the general concept are quite interesting and helpful towards the wider adoption of integrated pest management and the challenges. The quality is clearly very good.

 Some minor comments can be found below in order to further improve the quality of the manuscript and avoid confusion:

- In the abstract and introduction, it would have been nice to provide the number of stakeholders.
- Furthermore, the role of pesticides as an important part of IPM should be also mentioned in order to highlight the importance of their sustainable use.
- The eight principles of IPM could be also mentioned.
- In the methodology section, it would be helpful to specify the number of questions per survey.
- Furthermore, authors are encouraged to justify the number of the sample and to show that such a number (119 in total) can be adequate for some conclusions.
- The quality of the figures is very good, with only a recommendation to increase their size.
- As a general comment, it could be said that the authors could further highlight the novelty of the project and platform and discuss both the differences revealed between the several categories of stakeholders and the potential utility of their overall approach and project.

Is the study design appropriate and does the work have academic merit?

Yes

Is the work clearly and accurately presented and does it cite the current literature?

Yes

If applicable, is the statistical analysis and its interpretation appropriate?

Not applicable

Are all the source data underlying the results available to ensure full reproducibility?

Yes

Are the conclusions drawn adequately supported by the results?

Yes

Are sufficient details of methods and analysis provided to allow replication by others?

Yes

Reviewer Expertise:

Associate Professor of Agronomy & Weed Science

I confirm that I have read this submission and believe that I have an appropriate level of expertise to confirm that it is of an acceptable scientific standard.

# Response

**Published:** 2022-09-08

**Authors:** margherita furiosi

**Affiliations:** Università Cattolica del Sacro Cuore, Italy

The Authors are extremely grateful to Reviewers who took time to read and comment our paper. We addressed mostly all the comments and suggestion both directly in the text and in the rebuttal letter below (our responses in *italics*).

**#Reviewer 2** In general, the study is a comprehensive overview of the outcomes and activities within the IPMWorks H2020 project. The overall approach and the general concept are quite interesting and helpful towards the wider adoption of integrated pest management and the challenges. The quality is clearly very good.

 Some minor comments can be found below in order to further improve the quality of the manuscript and avoid confusion:

 In the abstract and introduction, it would have been nice to provide the number of stakeholders.

*Done.*

 Furthermore, the role of pesticides as an important part of IPM should be also mentioned in order to highlight the importance of their sustainable use.

*Done.*

 The eight principles of IPM could be also mentioned.

*Honestly, we believe that the link to the official EU website* *https://food.ec.europa.eu/plants/pesticides/sustainable-use-pesticides/integrated-pest-management-ipm_en* *and the citations of three reference papers is quite enough to give a comprehensive explanation to the reader.*

 In the methodology section, it would be helpful to specify the number of questions per survey. *Done.*

 Furthermore, authors are encouraged to justify the number of the sample and to show that such a number (119 in total) can be adequate for some conclusions.

*Actually, the total number of respondents were 343. Unfortunately the EU projects have some clear and strict timeline and deadlines so it was not possible to wait longer so to increase the number of respondents.*

 The quality of the figures is very good, with only a recommendation to increase their size.

*We followed ORE requirements, by the way it is possible to download the figures both as ppt and pdf.*

# Peer review: Reviewer response for version 1

**DOI:** 10.21956/openreseurope.15856.r29640

**Published:** 2022-08-16

**Authors:** Emilio Gil, Ramon Salcedo

**Affiliations:** 1 Unit of Agricultural Machinery Research, Department of Agri Food Engineering and Biotechnology, Universitat Politècnica de Catalunya, Barcelona, Spain

The manuscript gives an overview of the excellent and useful work done within IPMWORKS EU project. The idea, objective and results seem really interesting and useful for the improvement of implementation of IPM all around Europe.

**Abstract:**

- What do you mean by ‘internal and external stakeholders’?
- The authors indicate twice in the abstract that the survey was conducted in "multiple languages." I suggest that once is enough. It may also be interesting to point out the number of European countries through which the survey has circulated.
- Instead of saying "different stakeholders", I would say the number of stakeholders consulted.

**Introduction**

- IPM and its implementation became mandatory in EU since January 2014. So, theoretically, after 8 years of the entry in force, these practices should be already fully implemented all around Europe. Then, projects and actions as IPM Works and other mentioned in this article should not be needed. The problem is that, after 8 years of the mandatory success in EU of IPM has been very low. Authors should include some objective information about the results of conducted official audits of SUD implementation among EU members, and difficulties encountered on the implementation of IPM.
- Additionally, even if the introduction chapter is adequate, it seems that the use of Plant Protection Products within IPM program has been forgotten by the authors. PPP and its adequate and safe use is one of the measures included in IPM and there is a large list of EU projects, some of them similar to IPM WORKS including developed freely accessible platforms, to help farmers in the complex process of PPP application. I suggest authors should at least include some of these important actions conducted already in Europe.
- The problems mentioned ('resistance to agro-chemicals, reduction of natural enemies' and non-target organisms' population, and rise of chemical residues in final products') are absolutely true, but given the great efforts that are being made to reduce losses during pesticide applications, I suggest some additional comment in this regard (airborne spray drift, ground losses).
- "In Europe IPM became mandatory in 2014 (Directive 2009/128/EC)" I suggest linking this with the current European strategy "From Farm to Fork".
- "IPM is based on eight principles" - Could you summarize these 8 principles for us?
- I would say "Agricultural production inevitably becomes more complicated over time, as new innovations in plant breeding, machinery, application technology, and management systems require more time..."
- “whether”?
- "DSSs and other resources still have a limited uptake among farmers due to a range of actual and perceived barriers, such as farmers not being aware of what resources are available" I suggest adding that efforts are still being made from Europe to correct this, such as the H2020 Innoseta project or the BTSF courses of the European Commission.
- "Interactive" appears twice.
- What is the difference between 'training material' and 'resource evaluation material'?

**Methods**

- The sentence “Almost 90% of respondents were farmers, agronomist and researchers, minimizing the risk of bias” is unclear. How is the risk minimized?
- In the additional comments’ chapter, it has been written that “being a platform available across Europe, contents should be expressed in user’s native languages”. Of course, this is obvious and there are already different platforms available in different languages.
- How did you translate the tool into several languages? Who helped you?
- Given that farmers are the end user of this application (119), may the authors say where the responses have mainly come from (the 3 countries with the most surveys)? This is important, given the degree of heterogeneity within European agriculture.
- How many questions were there in total per survey?
- "Microsoft Excel was used to manage data and to create graphs and tables" - and did the authors not use any other additional software to analyze the statistics of the data, such as R or SSPS?

*Table 1: *

- 119 answers from farmers, considering the participation of 17 countries, seems a very poor number and, consequently, difficult to extract conclusions about the opinion of EU farmers. It represents an average of 7 farmers/country. This value cannot be acceptable to extract conclusions. The same comment can be applied for the rest of stakeholder’s groups.
- When the authors say "number of answers", do they mean the number of surveys? Or the number of questions answered?

**Results:**

- "Most of them expected it to be an interactive platform (40% of farmers and agronomists and 70% of researchers)" - That's almost double! Why do you think there is this difference?
- Figures 3-5. I don't quite appreciate how many different shades of blue there are. I have counted, if I am not mistaken, four. This is confusing to me, since the bars indicate the percentages of a survey that range from 1 to 5. I suggest a clearer bar system or at least include the blue scale from 1 to 5.
- I ask myself a question. Respondents indicated that they wanted a tool that above all else was simple, clear, in their native language, and with more images than text. However, this is already being implemented in most DSSs today. Why do these communication problems still occur between the latest advances in pesticide management and application and end users?

In general, there is too much text on the importance of survey and very little explanation about the platform itself. How the material has been organized, how users can easily (or not) find the information, which kind of materials are available, and other important practical information expected when a tool box platform is presented. Survey represents just the starting point but, in this article, it seems the core of the project.

 Figure 7 is the only practical information of the platform and, unfortunately, due to its size, it is nearly impossible to read it. Also, the same comment is applied to Figure 8.

 In the conclusions it has been written: “The toolbox will be an important tool for spread the adoption of IPM principles across Europe”. Does it mean that platform is still under development? If it is already available, this sentence should be removed and, of course, a clear and direct link to the platform should be provided.

Is the study design appropriate and does the work have academic merit?

Yes

Is the work clearly and accurately presented and does it cite the current literature?

Yes

If applicable, is the statistical analysis and its interpretation appropriate?

Not applicable

Are all the source data underlying the results available to ensure full reproducibility?

Partly

Are the conclusions drawn adequately supported by the results?

Yes

Are sufficient details of methods and analysis provided to allow replication by others?

Yes

Reviewer Expertise:

Full professor in Ag Engineering, focused in spray application technology and aspects related with environmental contamination

We confirm that we have read this submission and believe that we have an appropriate level of expertise to confirm that it is of an acceptable scientific standard, however we have significant reservations, as outlined above.

# Response

**Published:** 2022-09-08

**Authors:** margherita furiosi

**Affiliations:** Università Cattolica del Sacro Cuore, Italy

The Authors are extremely grateful to Reviewers who took time to read and comment our paper. We addressed mostly all the comments and suggestion both directly in the text and in the rebuttal letter below (our responses in *italics*).

**#Reviewer 1** The manuscript gives an overview of the excellent and useful work done within IPMWORKS EU project. The idea, objective and results seem really interesting and useful for the improvement of implementation of IPM all around Europe.

**Abstract** What do you mean by ‘internal and external stakeholders’?

*We meant stakeholders both directly involved in IPMworks and not*. 

 The authors indicate twice in the abstract that the survey was conducted in "multiple languages." I suggest that once is enough. It may also be interesting to point out the number of European countries through which the survey has circulated.

*The survey was conducted in four languages (English, French, Italian and Dutch): modified in the abstract. The Survey circulated across all Europe, but we got responses from 20 countries (see figure 1).*

 Instead of saying "different stakeholders", I would say the number of stakeholders consulted.

*The total number of respondents were 343, but not all of them were growers: we received replies also from researchers, agronomists, policy makers, and plant protection organizations … for these reasons we believe that “different stakeholders” is better describing the different sources. In any case, we adjusted the abstract in order to be much clearer. Please see also table 1 for more details.*

**Introduction** IPM and its implementation became mandatory in EU since January 2014. So, theoretically, after 8 years of the entry in force, these practices should be already fully implemented all around Europe. Then, projects and actions as IPM Works and other mentioned in this article should not be needed. The problem is that, after 8 years of the mandatory success in EU of IPM has been very low. Authors should include some objective information about the results of conducted official audits of SUD implementation among EU members, and difficulties encountered on the implementation of IPM.

*Actually, the aim of the research project IPMworks and the toolbox developed is exactly about overpassing difficulties of application and spread of IPM techniques*

  Additionally, even if the introduction chapter is adequate, it seems that the use of Plant Protection Products within IPM program has been forgotten by the authors. PPP and its adequate and safe use is one of the measures included in IPM and there is a large list of EU projects, some of them similar to IPM WORKS including developed freely accessible platforms, to help farmers in the complex process of PPP application. I suggest authors should at least include some of these important actions conducted already in Europe. 

*Added in the text.*

 The problems mentioned ('resistance to agro-chemicals, reduction of natural enemies' and non-target organisms' population, and rise of chemical residues in final products') are absolutely true, but given the great efforts that are being made to reduce losses during pesticide applications, I suggest some additional comment in this regard (airborne spray drift, ground losses).

*Added in the text.*

 "In Europe IPM became mandatory in 2014 (Directive 2009/128/EC)" I suggest linking this with the current European strategy "From Farm to Fork". 

*https://food.ec.europa.eu/horizontal-topics/farm-fork-strategy_en* *to be included in the modified text.*

 "IPM is based on eight principles" - Could you summarize these 8 principles for us?

*Honestly, we believe that the link to the official EU website* *https://food.ec.europa.eu/plants/pesticides/sustainable-use-pesticides/integrated-pest-management-ipm_en* *and the citations of three reference papers is quite enough to give a comprehensive explanation to the reader.*

 I would say "Agricultural production inevitably becomes more complicated over time, as new innovations in plant breeding, machinery, application technology, and management systems require more time..."

*Yes, corrected in the text.*

 “whether”? Typo, sorry.

*Corrected in the text.*

  "DSSs and other resources still have a limited uptake among farmers due to a range of actual and perceived barriers, such as farmers not being aware of what resources are available" I suggest adding that efforts are still being made from Europe to correct this, such as the H2020 Innoseta project or the BTSF courses of the European Commission.

*Added in the text.*

 "Interactive" appears twice. Corrected in the text What is the difference between 'training material' and 'resource evaluation material'?

*Clarified in the text.*

**Methods** The sentence “Almost 90% of respondents were farmers, agronomist and researchers, minimizing the risk of bias” is unclear. How is the risk minimized?

*The idea was to avoid the creation of a top-down proposed platform: having 90% of answers from farmers made us confident of a bottom-up approach. Better clarified into the text.* 

 In the additional comments’ chapter, it has been written that “being a platform available across Europe, contents should be expressed in user’s native languages”. Of course, this is obvious and there are already different platforms available in different languages.

*We agree.*

 How did you translate the tool into several languages? Who helped you? In IPMworks we are 35 Partners from all across EU. *Everyone helped.*

 Given that farmers are the end user of this application (119), may the authors say where the responses have mainly come from (the 3 countries with the most surveys)? This is important, given the degree of heterogeneity within European agriculture.

*We think that Figure 1 is quite clear.*

 How many questions were there in total per survey?

*10 questions, some multiple choice and some using Likert scale.*

 "Microsoft Excel was used to manage data and to create graphs and tables" - and did the authors not use any other additional software to analyze the statistics of the data, such as R or SSPS?

*No.*

*Table 1: *119 answers from farmers, considering the participation of 17 countries, seems a very poor number and, consequently, difficult to extract conclusions about the opinion of EU farmers. It represents an average of 7 farmers/country. This value cannot be acceptable to extract conclusions. The same comment can be applied for the rest of stakeholder’s groups.

*We can agree with this comment: nevertheless, EU funded Projects have a quite clear timeline and deadlines thus it was not possible to wait longer for obtaining more feedbacks. Moreover, yes, it is true that the farmers are the final “practical” users but more than often they are part of a network with other stakeholders (researchers, agronomists, retail organisations etc.) so the number to use is 343.*

 When the authors say "number of answers", do they mean the number of surveys? Or the number of questions answered?

*The number of surveys completed: clarified in the table. * 

**Results:** "Most of them expected it to be an interactive platform (40% of farmers and agronomists and 70% of researchers)" - That's almost double! Why do you think there is this difference?

*Most probably a researcher is much more aware of what an interactive platform is and which type of advantages can provide.*

 Figures 3-5. I don't quite appreciate how many different shades of blue there are. I have counted, if I am not mistaken, four. This is confusing to me, since the bars indicate the percentages of a survey that range from 1 to 5. I suggest a clearer bar system or at least include the blue scale from 1 to 5.

*The shades of blue are 5, unfortunately not all the five categories were always available (if no one scored “1” the darker colour is not present). A note has been added into the captions.*

 I ask myself a question. Respondents indicated that they wanted a tool that above all else was simple, clear, in their native language, and with more images than text. However, this is already being implemented in most DSSs today. Why do these communication problems still occur between the latest advances in pesticide management and application and end users? *We don’t have a definitive answer (unfortunately), nevertheless, most probably, it is due to the lack of “bridge” between research/innovation and practical agriculture. We hope that IPMworks, as well as many other initiatives, could shorten this gap. In general, there is too much text on the importance of survey and very little explanation about the platform itself. How the material has been organized, how users can easily (or not) find the information, which kind of materials are available, and other important practical information expected when a tool box platform is presented. Survey represents just the starting point but, in this article, it seems the core of the project.* *Actually, the platform is at the beginning of its life: it has been just structured and developed and it will be launched in October. In fact, this paper is about the development of the toolbox.*

 Figure 7 is the only practical information of the platform and, unfortunately, due to its size, it is nearly impossible to read it. Also, the same comment is applied to Figure 8.

*As previously commented, this is just a “preview” of the toolbox. Nevertheless, due to ORE requirements the screenshots are at the maximum possible quality. If you download the pdf of the paper the shape is better. Sorry for that.*

 In the conclusions it has been written: “The toolbox will be an important tool for spread the adoption of IPM principles across Europe”. Does it mean that platform is still under development? If it is already available, this sentence should be removed and, of course, a clear and direct link to the platform should be provided.

*Yes, the platform is under development and will be released according EC in October 2022. After the official launch (and if the Editorial Board of ORE agrees) we can add the running link for the Toolbox.*
